# Experiences, Perceptions, and Coping Patterns of Emergency Department Nurses with Occupational Stressors in Saudi Arabian Hospitals: Mixed-Method Study

**DOI:** 10.3390/healthcare10081504

**Published:** 2022-08-10

**Authors:** Majed M. Alruwaili, Fuad H. Abuadas, Phillip Maude, Alistair Ross

**Affiliations:** 1 Nursing Administration & Education Department, College of Nursing, Jouf University, Sakaka 72388, Saudi Arabia; 2 Community Health Nursing Department, College of Nursing, Jouf University, Sakaka 72388, Saudi Arabia; 3 La Trobe School of Rural Health, Violet Marshman Centre for Rural Health, La Trobe University, Melbourne, VIC 3086, Australia

**Keywords:** occupational stress, nursing, emergency department, coping, Saudi Arabia

## Abstract

Extended working hours, a complicated workplace environment, and engagement in numerous physical and psychological stressors have been associated with the stressful nature of the nursing profession. Only a few studies have provided some insight into workplace stress and coping strategies adopted by nurses in Saudi Arabia and neighboring countries. Therefore, this study utilized a mixed-method design to explore the numerous factors that lead to stress among emergency nurses, their experience and perception of stress, and the coping mechanisms they find useful. A survey containing four domains and 86 items was adapted, pilot tested, and validated. The quantitative phase recruited 296 nurses who returned completed questionnaires, and then 21 nurses were interviewed for the qualitative phase. In total, 89.5% (*n* = 265) of the participants were female, 51% (*n* = 151) were aged 20–29 years, 83% (*n* = 246) were non-Saudi nationals, 49% (*n* = 145) were married, and 82% (*n* = 245) had a bachelor’s degree. The most common causes of occupational stress were work overload, personnel shortages, and inadequate pay. The qualitative phase data revealed five primary themes, including increased workload, rising living costs despite equal compensation, and staff shortages as main stressors. In addition, the study found that praying and spending quality time with friends are the primary coping techniques among nurses. The study results contribute to a better understanding of nurses’ working conditions in the emergency department. Additionally, they may contribute to the development of policies and practice reforms to improve Saudi nurses’ well-being, health, and overall work experience.

## 1. Introduction

Occupational stress has become a widespread public health problem in recent decades [1,2]. Globalization and technical advances have significantly changed the workplace by presenting new forms of organizational work, labor relations, and employment patterns [3,4,5]. Despite this pervasive nature of increasing work-related stress, it is evident that health professionals experience significantly higher stress levels and multiple adverse effects compared with workers in other sectors [2,6]. Nurses represent the largest single occupational group in the healthcare sector [7,8,9]. However, nurses face significantly higher pressure levels than other occupational groups because of the broad range of tasks and responsibilities they assume for the effective functioning of any healthcare system [10,11]. There are compelling claims that the demanding nature of the nursing practice significantly contributes to increased staff turnover and a global shortage of nursing professionals [8,12,13]. According to a Japanese survey [14], the average monetary value of absenteeism due to occupational stress was 520$ per person, whereas presenteeism (loss of organizational productivity) costs 3055$ per person/annually [14]. Warren, et al. [15] report that the indirect cost of presenteeism in the United States has reached 36$ billion, with a 13% productivity loss. Similarly, head nurses in China assessed a 21% decrease in job productivity due to their subordinates’ presenteeism. In addition to the productivity loss caused by occupational stressors, a study conducted by Shan, et al. [16] found that it causes an 18% increase in the rate of patient falls and medical mistakes [16].

In developed countries, nurses face a lot of pressures (rapidly aging population, increasing rates of chronic disease, rising healthcare costs and patient expectations, high staff turnover, and the rising costs and consequences of clinical errors). The interplay of all these factors means that occupational stress has become an ever-increasing challenge for nurses and healthcare workers [3,17]. The capacity of nurses to manage all of these job-related stressors is determined by the individual nurse, their socio-cultural background, and their demands [18,19,20]. However, the literature indicates that factors influencing coping capacity include the source of the stress, the workplace conditions and situation, and the individual nurse’s stress perception [21,22]. According to the literature, the most prominent coping techniques identified by nurses were problem-solving, peer assistance, and avoidance [23]. On the other hand, nurses’ coping methods might be adaptive or maladaptive; they can even be a problem- or emotion-focused. [18,23].

The growing understanding of current evidence on job-related stressors suggests that the prevalence and consequences of occupational stress are worse among emergency department nurses because they are exposed to a complex and ever-changing work environment and high emotional stress [23,24,25]. Compared to nurses in other hospital departments, nurses in emergency departments work in an environment characterized by constant aggression and violence, high levels of suffering, and sometimes even patient death [23,25]. They work in a very dynamic environment where they often do not have the opportunity to build relationships or connections with their clients [23,25]. They must move very quickly from one client to the next, without time or opportunity to debrief or cope with the emotional or psychological toll of caring for patients in dire conditions [25]. Hogan et al. [26] pointed out in a study conducted to assess nurses’ coping with patient death and suffering that working in an emergency department can be physically and mentally exhausting. Undoubtedly, such a work environment may be even more demanding for most Saudi Arabian nurses [27,28], as they come from abroad and have to deal with various social and cultural hurdles. Hence, the decision to focus on the impact of occupational stress on emergency department nurses in Saudi Arabia, and the consequences for nurses, individually and collectively, and the Kingdom’s healthcare system. Unfortunately, there has been little research on the incidence and impact of occupational stress among Saudi Arabia’s nurses [27]. Consequently, this could affect the healthcare quality and healthcare system efficiency.

Some researchers have examined occupational stress incidence and impact in neighboring countries [27]. However, since Saudi Arabia relies on a large number of foreign nurses for providing healthcare, it is probable that the reasons for occupational stress are diverse and that the impact of these stressors on nurses and the healthcare system deserves further investigation [29,30]. Nowadays, nursing services are under a lot of stress and pressure as a result of the COVID-19 situation that is continually developing [31]. When nurses work in circumstances (such as COVID-19) with high workloads and few resources, their health and wellbeing may suffer due to increased workplace stress and physical and psychological symptoms [21,31]. Accordingly, studying professional stress and coping mechanisms among Saudi Arabian nurses may generate evidence-based policies and stimulate reforms that could improve working conditions. This study aimed to investigate the multiple factors associated with occupational stress among emergency department nurses of major hospitals in Saudi Arabia. It also investigated the nurses’ perception of stress and its effect on the quality of care and personal well-being. A mixed-method (sequential) design that combines quantitative and qualitative exploratory research data was recruited to address research objectives. Using a mixed-method design can lead to a deeper, stronger, and better understanding of how emergency nurses experience their daily work-related stressors and how they can cope with them. The quantitative data explored the multiple sources of occupational stress and the stress levels experienced or perceived by Saudi Arabian emergency department nurses. The qualitative data explored the perceptions and experiences of stress among Saudi Arabian emergency department nurses and their coping methods, because these variables can vary depending on the context and the culture.

## 2. Materials and Methods

### 2.1. Study Design and Patients

A sequential explanatory mixed-method research design was adopted in this study. This research design enabled the researcher to gather, analyze, and combine quantitative and qualitative data to better understand emergency department nurses’ occupational stress in Saudi Arabia. The study incorporated a structured questionnaire with an exploratory phenomenological approach that used semi-structured interviews to acquire open-ended qualitative data. A quantitative survey preceded the interview component. However, exploratory qualitative data may be more appropriate to understand how the emergency nurses feel work-related stress in their work and social lives, how work-related stress impacts their professional and public relationships, and what techniques they employ to cope with and reduce these negative consequences.

### 2.2. Sampling and Setting

The study was carried out in three large public hospitals’ emergency departments. The underlying rationale for choosing these hospitals is that emergency departments in these hospitals are among the busiest workplaces for nurses. This study adopted a total population sampling in which all nurses working in the target hospitals’ emergency departments was recruited. A total of 441 nurses were working in the target hospitals’ emergency departments, with 254, 124, and 63 nurses working in each. Criteria for participation in the study included at least 6 months of full- or part-time employment at the target hospital. On the other hand, the qualitative phase used a purposive sampling method. Of all nurses, 35 expressed interest in the study’s second qualitative phase and submitted their email addresses separately from the survey. A total of 21 nurses were interviewed for the qualitative phase.

### 2.3. Instrument

The study questionnaire was designed specifically to correspond with Lazarus and Folkman’s Transactional Theory of Stress and Coping, underpinning this study [32]. The theory is seen as a superior model for this study, since it emphasizes the importance of dynamic personal interaction with the environment in stress perception, expression, and coping. Furthermore, it is a more adaptable and dynamic framework for explaining the dynamic interplay of the nursing work environment, job demands, and personal coping resources. The researcher used the main themes of this stress theory to guide the development of the questionnaire to ensure that the data collected were consistent with the study’s objectives. The four parts of the research instrument are highlighted and illustrated in Table 1. A panel of nine experts, including academics and senior professional nurses, reviewed and validated the questionnaire, making necessary changes. The specialists were asked to score the relevance and clarity of the information. Each item had a range of 0.80–1, and the total scale content validity had a rate of 0.90, which met the minimum requirement. Cronbach’s alpha and Guttman split-half coefficient for the instrument were 0.878 and 0.959, respectively.

### 2.4. Data Collection

The RMIT Human Ethics Committee (SEHAPP 97-17, March 2018) and the Saudi Arabian Ministry of Health’s Institutional Review Board (H-01-R012, IRB log number: 17-474E) both examined and approved this study. With the support of the hospital nursing administration, the researcher organized a briefing meeting about the study to reach all working nurses. After the sessions, the survey was distributed to all emergency department nurses. The nurses were expected to return the completed survey to a safe and locked container in the emergency department (private area) within a 4-week period. A total of 441 questionnaires were distributed throughout the three hospitals.

**Figure 1:**
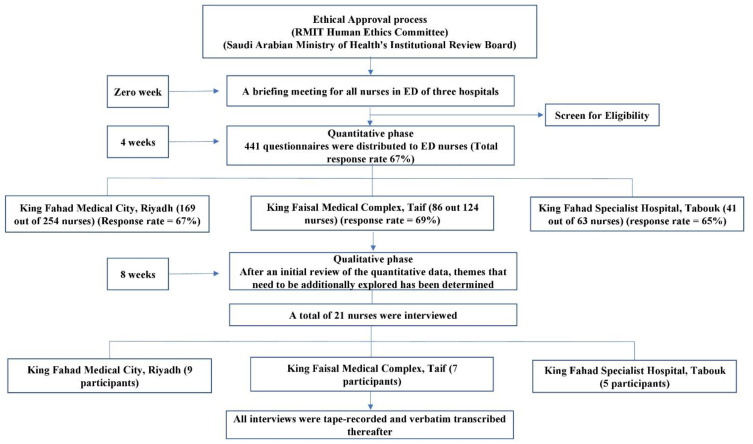
A schematic representation of the study protocol and data collection process.

After an initial review of the quantitative data, the researcher highlighted themes that can be additionally explored using qualitative data. These themes were utilized to build a guide for undertaking semi-structured interviews during the qualitative data collection phase. The interview guide included questions asking participants to explain what stress means to them and how they deal with stress during a typical workday. In this group of questions, nurses were asked to give specific examples of stressful events/circumstances and to indicate how often such situations or events occur at work. During their interviews, nurses also discussed important sources of stress, such as workload, work resources, and payment adequacy. A total of 21 nurses indicated their willingness to participate in the interviews. All interviews took place in quiet and secluded rooms within the emergency departments of the target hospitals. All interviews were conducted directly by the researcher and lasted between 35–50 min. All interviews were tape-recorded and verbatim transcribed thereafter. After all interviews were done, the researcher listened to all recordings to ensure that all participants were audible and clearly understood, which is essential for the quality of the data. Figure 1 provides a schematic representation of the study protocol and data collection process.

### 2.5. Statistical Analysis

Descriptive statistics (frequency distribution and percentage) were used to analyze the categorical demographic features of the nurses. Mean ranking was used to determine the most often stated sources and causes of occupational stress. In addition, ranking and mean rating was used to understand and explore the nurses’ perception of stress and coping strategies. After that, bivariate correlations and the chi-square test were used to assess the correlation of demographic factors with the sources of stress. Nvivo 11 software was used to manage and analyze the qualitative data phase. A content analysis was conducted to analyze the qualitative data in which common meanings were grouped into subthemes. After that, Similar subthemes were assembled to create overarching themes that finally constitute a central qualitative theme. The themes and subthemes identified in this analysis are presented in the results.

### 2.6. Ethical Considerations

The project received ethical approval from the Human Ethics Committee of RMIT (IRB approval no. SEHAPP 97 -17, Date: 7 March 2018) and the Ministry of Health, Saudi Arabia (H-01-R012, IRB log number: 17-474E). Several strategies were used to protect the participants’ confidentiality and anonymity. Each nurse was assigned a code number, and the researcher handled all submitted data electronically through a password-protected account after data entry. In addition, no name identification appeared in the study results, and all data were reported in an aggregate form.

## 3. Results

### 3.1. Quantitative Results

#### 3.1.1. Socio-Demographic Attributes of Participants

In total, 296 out of 441 emergency nurses returned the distributed questionnaire (response rate = 67%). They were mainly female and non-Saudi nationals; almost over two-thirds of the respondents in the study were female, which is typical of the nursing profession worldwide. In addition, over half of the participants were in their 20s, with nearly 90% being under the age of 40. Saudi nationals made up fewer than one-fifth (17%) of the nurses, while foreign nurses came mainly from India (41.2%) and the Philippines (39.8%). The most prevalent qualification was a bachelor’s degree. A diploma in nursing followed in second place by a wide margin. Participants in their 20s were much more likely to hold a bachelor’s and a master’s degree, while participants in their 50s were much more likely to hold a diploma as their highest education level.

The emergency nurses in this study had a range of 1 to 11 years of nursing experience. More than half of those participants have been nurses for 6 years or less. However, a substantial proportion had more than 11 years of professional experience, ensuring that the sample has a solid balance of working experience. There is a similar mix of working experience in the emergency department. More than 78% of respondents have worked in the emergency department for 6 years or less, and nearly 5% have more than 10 years of experience in the emergency department. The participants’ socio-demographic characteristics are illustrated in Table 2.

#### 3.1.2. Occupational Sources of Stress among Emergency Department Nurses

The perceived occupational stressors were assessed based on a questionnaire consisting of 57 items measured on a 7-point Likert scale, subdivided into five domains, adapted from Lu, Sun, Hong, Fan, Kong and Li [23] and Moustaka and Constantinidis [12]. The most stressful sub-domains as perceived by emergency nurses were “workload and time assignment” (M = 3.4, SD = 1.6), “Working conditions and resources” (M = 2.7, SD = 1.3), and “Nurses specialty and work” (M = 2.68, SD = 1.28). The averages of these sub-domains show that the study sample experienced moderate stress. Items in the workload sub-domain were reported as the most stressful sources, highlighting the role of workload as a work-related stressor among emergency department nurses. In contrast, the least stressful sub-domains as perceived by emergency nurses were “patient care” (M = 2.53, SD = 1.26), and “management and interpersonal relationship” (M = 2.14, SD = 1.34). It is interesting to note that despite the various multinational structure of nursing staff in emergency departments, management and interpersonal factors ranked last among the causes and sources of stress.

To gain a better understanding regarding important sources of stress for nurses at work, the average of participants’ responses was calculated for each item in each sub-domain. The descriptive results for the items in the sub-domains showed that item “ The amount of paperwork I have to do is excessive.” had the highest mean score (M = 3.9), a workload-related factor. In contrast, the lowest mean was obtained for items related to management and interpersonal aspect. Table 3 illustrates the most and least stressful items for emergency department nurses.

To better understand how participants’ attributes influence the level of reported stress, responses on the 6-point scale to all items on the survey were grouped into three categories—high stress (5 and 6), low stress (0, 1, and 2), and moderate stress (3 and 4). Spearman’s rank correlation coefficients were calculated to check associations between participants’ attributes and the stress level in each of the five domains of the nursing job as categorized in the scale. There were significant correlations between emergency department working (years of experience) and the degree of stress indicated from items in the domain of ‘nurse specialty’ (X2 = 7.04, Spearman r = 0.13, *p* < 0.05) and ‘patient care’ (X2 = 12.06, Spearman r = 0.17, *p* < 0.01). Participants with less than 2 years of experience report considerably greater stress from elements in the nursing specialty domain and patient care, whereas those with 11 years of experience or more report significantly lower stress. Similarly, there were significant correlations between the level of education and the degree of stress indicated from items in all domains. Participants with a higher level of education reported a significantly higher level of stress. Table 4 illustrates the significant correlations.

#### 3.1.3. Perceptions and Experience of Stress

More than half of the nurses in this research (52.4%) said that they felt upset ‘sometimes’ or ‘fairly often’ because of something unexpected, while another 12.8% say they’ve been upset ‘very often.’ Likewise, over 53% of study participants said they ‘sometimes’ or ‘fairly often’ feel powerless over crucial matters. More importantly, over 60% of participants say they have been worried and stressed ‘sometimes’ or ‘fairly often,’ with another 18.5% saying they have felt nervous and stressed ‘very often.’ These findings indicate that the nurses in this study report considerable amounts of stress in their job and daily lives (Table 5).

Furthermore, the perceived stress scale [12] allows for the calculation of stress scores for research participants by inverting responses (that is, 0,1,2,3,4 to 4,3,2,1,0) to positively phrased items (4, 5, 7, 8) and then aggregating responses for all items in the scale. The potential score ranges from 0 to 40, with 0–13 indicating low perceived stress, 14–26 indicating moderate stress, and 27–40 indicating high stress. Most emergency nurses (90.2%, *n* = 267) reported moderate stress scores (ranging from 14 to 26), with just roughly 5% (*n* = 16) expressing severe stress levels. A small proportion (4.4%, *n* = 13) of nurses reported perceived stress scores ranging from 0 to 13, suggesting a low stress level.

#### 3.1.4. Coping with Stress

Two open-ended questions were used to investigate the most stressful part of a nurse’s job (sources of stress). About 30% of the respondents (*n* = 91) did not complete this survey section. However, 36% (*n* = 74) and 34% (*n* = 70) of the total respondents who completed this survey section reported insufficient staff and workload/patient-to-nurse ratio, respectively. Respondents were also asked to choose which coping techniques they would employ “when experiencing a poor day at work”. Prayer was the most prevalent coping strategy among participants, with 40.8% saying they ‘pray’ when they’re having a bad day. Other common coping mechanisms mentioned by the nurses in this study were speaking with a friend (18%), sleeping (10.2%), and listening to music (8.3%). The final section of the survey asked nurses to describe what factors or adjustments they believe would make working less stressful in the emergency department. Even though around 30% of respondents did not complete this section of the survey, the results revealed that over 65% of those who responded believed that having enough nursing staff on duty is a critical improvement needed to alleviate occupational stress. Furthermore, nearly 15% of nurses believed that lowering workload might reduce perceived stress, while another 7% assumed that better staff collaboration is essential to reduce stress.

### 3.2. Qualitative Results

Participants in this second phase (17 females and 4 males) shared the same demographic characteristics as those in the previous phase: they were predominantly female, young (under 40 years old), mostly foreigners, and had 2 to 10 years of professional experience. In total, 25 primary themes emerged from qualitative data analysis, grouped under five (5) major thematic categories.

The first theme focuses on nurses’ experience of stress and their perception of work-related stress prevalence. It highlights how nurses in the emergency department are stressed by every aspect of their job, from dealing with a steady flow of patients to addressing the needs of patients and their families. The second theme provides a deeper insight into nurses’ perceptions of the severity and seriousness of workplace stress. It highlights the influence of the work environment and context, indicating that the levels of stress reported by nurses are not just a result of their job activities but also of the work environment and organizational issues. This theme illustrates that a lack of adequate pay, lack of sufficient work equipment, lack of promotion opportunities, and poor communication with hospital management contribute to an increased level of stress among nurses. The third theme focuses on the effects and consequences of stress on nurses’ health and professional performance. Nurses interviewed believe that their high levels of stress at work impact their ability to offer safe and high-quality treatment. Some nurses said emphatically that it is unreasonable to expect good-quality treatment while they are responsible for a steady flow of high patient load, particularly in the face of long-lasting nursing and non-nursing staffing shortages.

The fourth theme focuses on how nurses help and support each other. Some nurses described the helping relationship as having become like family members. The fifth theme emphasizes the wide range of coping mechanisms used by nurses to combat the effects of stress on their health and professional performance. Despite the presence of certain maladaptive coping mechanisms such as unhealthy eating, smoking, and avoidance, the data revealed that praying, spending more time with family, having a break, exercising, and other positive coping strategies were the most common. This highlight the nurses’ dedication to their jobs, persistence, and a wide range of coping skills and techniques that they use to be productive and preserve their well-being despite high stress levels.

## 4. Discussion

The goal of this study was to explore which job-related factors emergency department nurses consider to be the most prominent source of occupational stress. According to the quantitative data in this study, workload, time management, and working environment were the most significant stress sources for nurses in the emergency department. This is consistent with previous studies conducted in other nations or cultural contexts [10,36,37,38,39,40,41,42] and further supports the argument that workload is top on the list of nurses’ occupational stressors worldwide. ‘Heavy workload,’ according to Zhou and Gong [38], is the primary source of stress for nurses working in Chinese hospitals, and ‘workload,’ according to Godwin, Suuk, and Selorm [39], is reported as stressful among nurses working in Ghana. Likewise, in their five-country study, Glazer and Gyurak [40] found workload as the top source of stress for nurses. Jones, Hocine, Salomon, Dab, and Temime [41] and Stimpfel, Brewer, and Kovner [42] used skipped breaks as a predictor of high workload in emergency and critical care units. Over a three-shift period, Jones [41] discovered that more than 40% of emergency department nurses omitted at least one break to meet their workload, while Stimpfel [42] revealed that more than 50% of the emergency nurses in their research did not take any breaks during their shift. In contrast to previous similar studies, items in the “management and interpersonal relationships” domain in this study were not reported as substantial stress sources for nurses. For example, several studies such as [43,44,45,46] suggest that working in a multinational environment, as most of the nurses in this research do, can be a considerable source of stress.

Furthermore, a comparison of demographic information and perceived stress levels reveals that nurses with more extensive professional experience are often more likely to have a lower level of stress than nurses with fewer than 5 years of work experience. This also suggests that nurses with more work experience are less concerned about being injured or disabled, but they are concerned about making clinical errors or triggering patients’ discomfort during treatment. These results were consistent with the study by Yim, et al. [47], which discovered that nurses’ psychological capital evaluations improved with years of professional experience and showed a substantial negative correlation with reported occupational stress. So, one possible explanation is that nurses who have more extended working time experience have built greater stability and resilience and, hence, do not worry as much about the likelihood of harm, thus reporting lower levels of stress [47]. Combining quantitative and qualitative data revealed that workload is a multidimensional entity, and such an understanding is required and vital for devising effective interventions or organizational changes to enhance nurses’ working circumstances.

According to the quantitative data, the majority of the nurses in the research report a moderate level of stress, with roughly 5% reporting “severe” stress levels. Similarly, qualitative data revealed that the critical effects of professional stress stem from a persistent experience of being overwhelmed and having little control over time, work, or their daily routine. These data highlight numerous physical, psychological, and intellectual effects of occupational stress on emergency department nurses. Such health consequences have garnered considerable attention in the literature [8,36,48]. Adriaenssens, De Gucht and Maes [36] found that depression and anxiety among nurses could be predicted based on perceived stress levels. Similarly, Chiang and Chang [8] found a correlation between the incidence of depression among nurses and their reported stress levels. Furthermore, several studies [8,36,43,48,49,50,51] revealed links between perceived occupational stress, caregivers’ emotional and intellectual health, and quality of care.

According to Burgess, et al. [33], coping strategies can be roughly described as attempts to modify the circumstances of the issue to minimize perceived stress (problem-focused coping) or attempts to reform the emotional implications of the reality (emotion-focused coping) [33]. The current study shows that emergency department nurses utilize a variety of coping mechanisms that fall into both problem and emotional-focused coping methods. The most prominent coping mechanisms were prayer, speaking with friends/family, sleeping in, and playing music. Praying as an emotional coping method contradicts the practical, problem-focused coping strategies frequently reported in Western countries [24,26,39,40,46]. However, unpacking the qualitative data shows that nurses think that spending time in prayer offers a sense of tranquility and helps to ease stressed thoughts and feelings; hence, it may be viewed as a positive emotional coping approach.

Stepping beyond prayer, speaking to coworkers about difficulties and how to solve them, and falling asleep more were rated second and third on favored coping strategies, respectively. These positive coping methods are comparable to the prevalent coping techniques reported by care providers in other nations, such as the results of Godwin, Suuk, and Selorm [39] from nurses in Ghana or McCarthy, et al. [52] from nurses in Ireland. Furthermore, several studies have identified and emphasized the significance of ‘social support’ for nurses as a critical and effective strategy for reducing stress and its harmful effects [24,53,54,55]. According to Crilly, et al. [56], increasing years of professional experience is positively connected with healthier and more effective coping abilities, implying that young and inexperienced nurses are more prone to using emotional, maladaptive coping mechanisms. This explanation supports the outcomes of our study, which found that nurses with more work experience had lower perceived stress than younger nurses.

### 4.1. Strengths and Limitations

One of the study’s key strengths is that the nurses who participated came from various settings. However, the recruited nurses were from a hospital’s tertiary and secondary care levels. This paucity of rural and primary care perspectives may imply that the recorded perspective does not reflect nurses’ experience at the rural and primary care levels. In addition, the low response rate (67%) is considered to be another limitation in this study, given the busy nature of the nursing profession in the emergency department.

### 4.2. Implications to Nursing Practice

There is no doubt that there is a shortage in nursing worldwide. However, this study revealed that nurses require greater support in dealing with their caseload and that caregivers’ well-being should be taken into account when distributing or allocating cases. According to the findings of this study, the relative efficacy of workload redistribution among nurses suggests that nursing leadership in hospitals could establish an efficient and sustainable strategy to ensure that all nurses can manage their patients. Furthermore, having breaks for recreation and relaxation are essential components of resilience that nurses will need to cope successfully with job stress. Moreover, nursing leadership should assist and encourage social connection among nurses as an essential coping source.

## 5. Conclusions

The combined qualitative and quantitative data revealed how awareness and experiences of everyday work practices shape stress perception. The quantitative part of this study revealed staff shortages, nurses’ workload, and dissatisfaction with financial incentives as the major sources of stress in the emergency department. These data are amply supported by qualitative findings, which reveal the wide-ranging impacts of occupational stress on nurses’ performance, health, and well-being. Unlike previous research, this study discovered that conservative coping techniques such as prayer and talking with colleagues were the most common among emergency department nurses. This study also highlighted how the presence or absence of opportunities for social engagement affects the coping techniques employed by nurses to minimize the burden of work-related stress on their well-being and quality of care.

## Figures and Tables

**Table 1:** Structure of research instrument.

Section	Domain	Author	Description	Items
1	Sociodemographic characteristics	Developed by researcher	Close-ended multiple-choice and Yes/No questions	9
2	Sources of Stress	Adapted from Lu, Sun, Hong, Fan, Kong and Li [23], and Moustaka and Constantinidis [12]	57 items were measured on a 7-point Likert scale, subdivided into five domains:	57
			a. specialty and work (13 items)	
			b. workload (8 items)	
			c. working conditions and resources (12 items)	
			d. patient care (12 items)	
			e. management and interpersonal relationships (12 items)	
3	Perception of stress	Adapted from Perceived Stress Scale by Cohen, et al. [33]	10 items measured on a 5-point Likert scale (4 = very often, 3 = fairly often, 2 = sometimes, 1 = amost never, and 0 = never)	10
4	Coping methods	Adapted from Balducci, et al. [34] and McCoy [35]	Open-ended, fill in the blanks, close-ended multiple choice questions	9

**Table 2:** Socio-demographic characteristics of participants (*n* = 296).

Variables	*n*	%
Age		
20–29	151	51
30–39	114	38.5
>40	31	10.5
Gender		
Female	265	89.5
Male	22	7.4
Prefer not to say	9	3.1
Educational level		
Diploma	43	14.5
Bachelor’s degree	245	82.8
Master’s degree	8	2.7
Nationality		
Saudis	50	16.9
Filipino	118	39.8
Indian	122	41.2
Pakistani	2	0.7
others	4	1.4
Marital Status		
Single/Divorced	151	51
Married	145	49
Do you have children		
Yes	121	40.9
No	175	59.1
General Nursing Work Experience		
Less than 2 years	24	8.1
2–6 years	135	45.6
7–10 years	95	32.1
>11 years	42	14.2
ED Work Experience		
Less than 2 years	124	41.9
2–6 years	108	36.5
7–10 years	50	16.9
>11 years	14	4.7

ED: Emergency department.

**Table 3:** Ranking of perceived stress sources (*n* = 296).

Items	Total Rating	Mean	Rank
Excessive amount of paper work	1155	3.90	1st
Staff shortage	1134	3.83	2nd
Feeling tired on shift	1060	3.58	3rd
Providing Care for many patients	1049	3.54	4th
Adequate Payment and reward	1029	3.48	5th
**Last five items**			
Worry about any nursing procedures that may cause the pain	576	1.95	
Accomplishment of my job-related goals	549	1.85	
relationship with other nurses in this department is adversely affected my job	548	1.85	
I like the people I work with	511	1.73	
lack the skills/knowledge to provide effective patients education	509	1.72	

**Table 4:** Correlation coefficients between participants’ attributes and the level of stress in each of the five domains.

		Nurse Specialty and Work			Workload and Time Assignment			Working Conditions and Resources			Patient Care			Management and Interpersonal Relationships		
Participants Attributes		L	M	H	L	M	H	L	M	H	L	M	H	L	M	H
Gender	Male	5	15	2	4	11	7	5	12	5	3	13	6	9	10	3
	Female	78	146	41	59	100	106	86	136	43	107	123	35	142	98	25
		ρ (rs) = 0.04			ρ (rs) = 0.02			ρ (rs) = 0.057			ρ (rs) = 0.11			ρ (rs) = 0.11		
Marital Status	Single/divorced	37	88	26	29	60	62	38	87	26	48	82	21	75	63	13
	Married	47	79	19	34	57	54	55	66	24	65	59	21	80	50	15
		ρ (rs) = −0.08			ρ (rs) = −0.078			ρ (rs) = −0.13 *			ρ (rs)= −0.09			ρ (rs) = −0.02		
Age	20–29	39	86	26	27	58	66	44	82	25	57	72	22	76	60	15
	30–39	35	65	14	30	47	37	40	56	18	47	54	13	60	44	10
	>40	10	16	5	6	12	13	9	15	7	9	15	7	19	9	3
		ρ (rs) = −0.05			ρ (rs) = −0.06			ρ (rs) = 0.001			ρ (rs) = 0.04			ρ (rs) = 0.05		
Level of education	Diploma	22	19	2	19	12	12	25	14	4	27	12	4	32	9	2
	Bachelor	61	141	43	43	102	100	67	132	46	82	126	37	117	102	26
	Master	1	7	0	1	3	4	1	7	0	4	3	1	6	2	0
		ρ (rs) = 0.19 **			ρ (rs) = 0.18 **			ρ (rs) = 0.19 **			ρ (rs) = 0.14 *			ρ (rs) = 0.12 *		
General Nursing Work Experience	˂2 years	11	6	7	6	12	6	8	13	3	8	12	4	12	10	2
	2–6 years	34	82	19	23	50	62	38	74	23	50	65	20	69	49	17
	7–10 years	26	54	15	21	40	34	29	47	19	33	49	13	45	42	8
	>11 years	13	25	4	13	15	14	18	19	5	22	15	5	29	12	1
		ρ (rs) = −0.03			ρ (rs) = −0.07			ρ (rs) = −0.05			ρ (rs) = −0.08			ρ (rs) = −0.10		
ED Work Experience	˂2 years	44	64	16	29	48	47	64	63	15	58	53	13	71	44	9
	2–6 years	27	65	16	26	40	42	31	53	24	40	51	17	56	39	13
	7–10 years	10	29	11	7	23	20	12	30	8	10	31	9	20	24	6
	>11 years	3	9	2	1	6	7	4	7	3	5	6	3	8	6	0
		ρ (rs) = 0.13 *			ρ (rs) = 0.08			ρ (rs) = 0.10			ρ (rs) = 0.17 **			ρ (rs) = 0.07		

L—low stress; M—moderate stress; H—high stress; ρ(rs)—Spearman’s correlation coefficient; * *p* < 0.05, ** *p* < 0.01.

**Table 5:** Perception of stress descriptive statistics (frequency and percentage) (*n* = 296).

In the Last Month, How Often Have You	**Never, % (*n*)**	**Almost Never, % (*n*)**	**Sometimes, % (*n*)**	**Fairly Often, % (*n*)**	**Very Often, % (*n*)**
been upset because of something that happened unexpectedly	41(13.9)	62(20.9)	95(32.1)	60(20.3)	38(12.8)
felt that you were unable to control the important things in your life?	43(14.5)	68(23.0)	108(36.5)	50(16.9)	27(9.1)
felt nervous and “stressed”?	27(9.1)	36(12.2)	104(35.1)	74(25.0)	55(18.5)
felt confident about your ability to handle your personal problems?	36(12.2)	47(15.9)	103(34.8)	70(23.6)	40(13.5)
felt that things were not going your way?	35(11.8)	44(14.9)	134(45.3)	60(20.3)	23(7.7)
found that you could not cope with all the things that you had to do?	52(17.6)	56(18.9)	117(39.5)	50(16.9)	21(7.1)
been able to control irritations in your life?	28(9.5)	49(16.6)	125(42.2)	72(24.3)	22(7.4)
felt that you were on top of things?	54(18.2)	54(18.2)	124(41.9)	49(16.9)	15(5.1)
been angered because of things that were outside of your control?	41(13.9)	58(19.6)	104(35.1)	64(21.6)	29(9.8)
felt difficulties were piling up so high that you could not overcome them?	39(13.2)	64(21.6)	112(37.8)	55(18.6)	26(8.8)

## Data Availability

Not applicable.
